# Chitin Nanowhisker Aerogels

**DOI:** 10.1002/cssc.201200717

**Published:** 2013-01-18

**Authors:** Lindy Heath, Lifan Zhu, Wim Thielemans

**Affiliations:** [a] School of Chemistry, University of NottinghamUniversity Park, Nottingham NG7 2RD (UK); [b] Process and Environmental Research Division, Faculty of Engineering, University of NottinghamUniversity Park, Nottingham NG7 2RD (UK)

**Keywords:** gels, mesoporous materials, nanostructures, renewable resources, supercritical CO_2_

## Abstract

Chitin nanowhiskers are structured into mesoporous aerogels by using the same benign process used previously in our group to make cellulose nanowhisker aerogels. The nanowhiskers are sonicated in water to form a hydrogel before solvent-exchange with ethanol and drying under supercritical CO_2_ (scCO_2_). Aerogels are prepared with various densities and porosities, relating directly to the initial chitin nanowhisker content. scCO_2_ drying enables the mesoporous network structure to be retained as well as allowing the gel to retain its initial dimensions. The chitin aerogels have low densities (0.043–0.113 g cm^−3^), high porosities (up to 97 %), surface areas of up to 261 m^2^ g^−1^, and mechanical properties at the high end of other reported values (modulus between 7 and 9.3 MPa). The aerogels were further characterized by using X-ray diffraction, BET analysis, electron microscopy, FTIR, and thermogravimetric analysis. Characterization showed that the rod-like crystalline nature of the nanowhiskers was retained during the aerogel production process, making the aerogel truly an assembled structure of chitin nanocrystals. These aerogels also showed the lowest reported shrinkage during drying to date, with an average shrinkage of only 4 %.

## Introduction

Aerogels are unique materials that display unusual and highly desirable properties such as low densities, high porosities, high internal surface areas, and low heat conductivity.^2^ As a result, aerogels find application in areas such as catalysis,^3^ thermal insulation,^4^ drug delivery,^5^ gas storage,^6^ liquid absorption, and space and particle research.4b, 7 Organic aerogels, specifically aerogels made from polysaccharides such as cellulose and chitin, are of particular importance because they utilize renewable feedstocks.

After cellulose, chitin is the second most abundant biopolymer in the world. Chitin is composed of β-(1,4)-linked chains of *N*-acetyl-d-glucosamine and acts as a structural polymer in living organisms such as shellfish, microorganisms, and insects. Crab and shrimp shells are the most common source of chitin.^8^ Analogous to cellulose, chitin consists of both crystalline and amorphous domains. The amorphous domains can be hydrolyzed to release the crystalline segments, so-called chitin nanowhiskers, by using an acid hydrolysis procedure.^9^ The excellent intrinsic properties of chitin, for example, biocompatibility, non-toxicity, renewability, and biodegradability,^10^ result in chitin finding application in a wide variety of areas, such as medicinal and pharmaceutical applications,^11^ cosmetics,^12^ and the treatment of industrial pollutants.11a, 13 Chitin also displays excellent wound-healing characteristics and has been used in applications such as wound dressing materials and tissue engineering.11e, 14

Previously, chitin aerogels have been prepared by using the dissolution of chitin followed by regeneration in solvent, typically ethanol. Chitin dissolution systems include *N,N*-dimethyl-acetamide (DMAc)/lithium chloride (LiCl),^15^ 1-butyl-3-imidazolium acetate,^16^ and NaOH–urea solutions.^17^ Chow et al. reported chitin aerogels from a DMAc/LiCl solvent system with a high density of 8.02 g cm^−3^ and an extremely low porosity of 9.8 %.15a Tsioptsias and co-workers used the same solvent system to produce aerogels with densities in the range 0.125–0.226 g cm^−3^, porosities of 84.5–91.5 %, and Brunauer–Emmett–Teller (BET) surface areas of 220–363 m^2^ g^−1^.15b Silva et al. dissolved chitin in an ionic liquid, 1-butyl-3-imidazolium acetate, to produce aerogels with densities of 0.039–0.063 g cm^−3^, porosities of 84.1–90.2 %, BET surface areas between 108–145 m^2^ g^−1^, and pore volumes of 0.22–0.30 cm^3^ g^−1^.^16^ More recently, chitin aerogels made from the dissolution of chitin using 11 wt % NaOH–4 wt % urea aqueous solutions have been reported. These aerogels show similar low densities and surface areas up to 366 m^2^ g^−1^.17c In a different approach, Nogi et al. reported a chitin aerogel made from chitin nanofibers by using a freeze-drying technique resulting in a surface area of 260 m^2^ g^−1^.^18^ The different production procedures for these aerogels provided diverse structures and properties, allowing the materials to find applications in many areas.

To produce high-end materials by using cheap, renewable resources we have applied a similar technique to our previous work to obtain novel aerogels from chitin nanowhiskers. We were the first group to report the production of cellulose aerogels through the aqueous assembly of cellulose nanowhiskers.^1^ Several other groups have also reported cellulose nanoparticle aerogels using nanofibers as the starting material.^19^ For example, Pääkkö and co-workers produced aerogels from microfibrillated cellulose obtained by using a mild enzymatic hydrolysis and mechanical homogenization process. The nanofibers are flexible micrometer-long and partly amorphous fibrils, whereas cellulose nanowhiskers obtained by acid hydrolysis yield rigid, highly crystalline whiskers with lengths on the order of several hundreds of nanometers. The aerogels produced by Pääkkö and co-workers had low densities of 0.02 g cm^−3^, high porosities of 98.7 %, and a small shrinkage upon drying of around 7 %. However, these aerogels also displayed low surface areas of 70 m^2^ g^−1^, due to significant cellulose nanofiber aggregation during the freeze-drying process. Our aerogel production technique relied on the ability of the rigid rod-like whiskers of cellulose to form percolated hydrogen-bonded networks when dispersed by using forces with a similar order of magnitude as hydrogen bonds (4–50 kJ mol^−1^).^20^ We now successfully apply this technique to chitin nanowhiskers to produce the first reported chitin nanowhisker aerogels. Chitin hydrogels were first formed by dispersing the chitin nanowhiskers in water above the percolation threshold (above 40 mg mL^−1^) using a low-power ultrasoni-cation bath. Similar to the results observed for the cellulose nanowhisker aerogels, the ultrasound treatment did not alter or damage the crystalline structure of the chitin nanowhiskers. The hydrogels were solvent-exchanged with ethanol to give an alcogel before being dried by using scCO_2_. Aerogels produced in this manner displayed the expected high porosities and low densities.

## Results and Discussion

**Figure 1:**
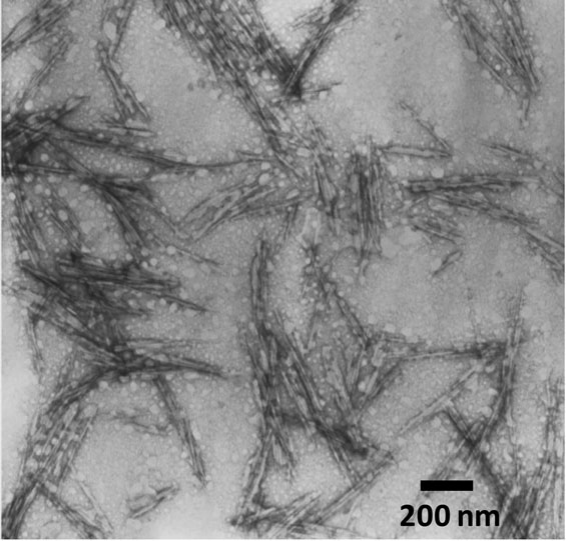
TEM image of chitin nanowhiskers derived from crab shells.

Figure 1 shows a transmission electron microscopy (TEM) image of chitin nanowhiskers negatively stained with a uranyl acetate solution prior to imaging. The chitin nanowhiskers have a rod-like morphology comparable to cotton-derived cellulose nanowhiskers and also align in bundles along their longitudinal axis because of hydrogen-bonding interactions. These interactions have already been shown to enable chitin nanowhiskers to form stable, gelled networks in aqueous conditions.^21^

Chitin nanowhisker hydrogels were constructed by the self-assembly of chitin nanowhiskers in water at concentrations above the percolation threshold. Increasing amounts of freeze-dried chitin nanowhiskers (from 40 mg to 120 mg) were added to 1 mL deionized water in a sonication bath for 30–45 min (Table 1). The energy provided by the sonication caused the nanowhiskers to disperse within the 1 mL volume, forming a 3D percolated network through interactions between surface C=O, N–H, and hydroxyl groups. At chitin nanowhisker contents below 40 mg mL^−1^, a dispersion was formed, but not a gel. In agreement with previous work on cellulose nanowhiskers reporting difficulties to fully redisperse freeze-dried nanowhiskers,^22^ these low-concentration dispersions did show some sedimentation over time due to nanowhisker aggregation. On the other hand, we use very dilute stable dispersions of nanowhiskers during freeze-drying to minimize nanowhisker interactions rather than utilizing the freeze-dried films used by Dong and Gray.^22^ It can thus be anticipated that there will be some aggregation within the percolated gel formed using the ultrasonication bath. The sonication bath provides forces with a similar order of magnitude to hydrogen-bonds (4–50 kJ mol^−1^),^20^ allowing the rearrangement and formation of hydrogen-bonds between the rigid rod-like chitin nanowhiskers, to form the 3D network throughout the aqueous volume. Once we obtained the hydrogels, a four-day solvent exchange with ethanol followed by drying for 6 h with scCO_2_ yielded fully dry aerogel monoliths.^1^ Little shrinkage was observed during the ethanol solvent exchange and scCO_2_ drying of the chitin aerogels.

**Table 1:** Quantities of chitin nanowhiskers used to produce the various aerogels

Chitin nanowhiskers [mg]	Deionized water [mL]	Sonication time [min]
40	1	45
50	1	45
60	1	45
70	1	45
80	1	45
90	1	45
100	1	30
120	1	30

**Figure 2:**
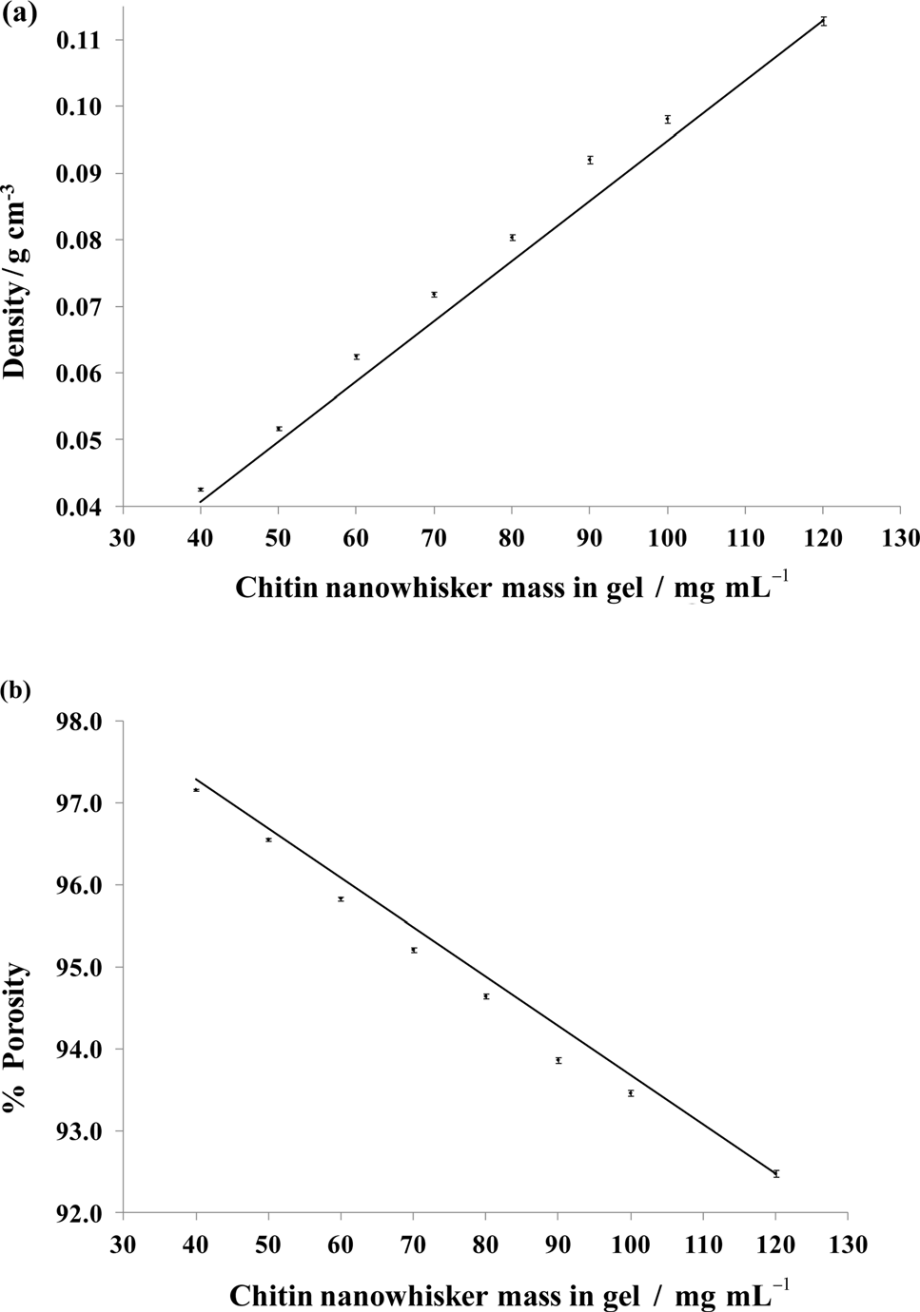
(a) Aerogel density in g cm^−3^ and (b) porosity (%) as a function of original chitin nanowhisker content in the hydrogel. The lines represent theoretical values if no shrinkage during drying occurs and the hydrogel forms in the full 1 mL water volume.

The density of the aerogels increased as the initial chitin nanowhisker mass in the aqueous 1 mL volume used for gelation increased. Figure 2 a shows variation between 0.043 and 0.113 g cm^−3^. The densities achieved for these aerogels were similar to the densities obtained for our previously reported cellulose nanowhisker aerogels (0.078–0.155 g cm^−3^), when comparing gels containing the same weight of nanowhiskers (chitin range 40-120 mg mL^−1^, reported cellulose range 80–160 mg mL^−1^).^1^ As expected, with an increase in aerogel density, there was an associated decrease in porosity [Equation (1)] from 97 % to 92 % (Figure 2 b), with values again similar to cellulose nanowhisker aerogels of the same nanowhisker content. The theoretical density and porosity were calculated assuming that the hydrogel forms in the 1 mL water volume and that there is no shrinkage during drying (indicated by the lines in Figure 2 a and 2 b). The average difference between the experimental and theoretical values for density and porosity for the chitin nanowhisker aerogels is 4 %, indicating extremely low shrinkage during drying. This value indicates gel formation in the 1 mL water volume and confirms the visual observation that there was little shrinkage during drying. This percentage shrinkage is also comparable to the average 6.5 % shrinkage observed for the cellulose nanowhisker aerogels.^1^ The close correlation between the experimental and theoretical density and porosity means that these properties can be accurately predicted.

**Figure 3:**
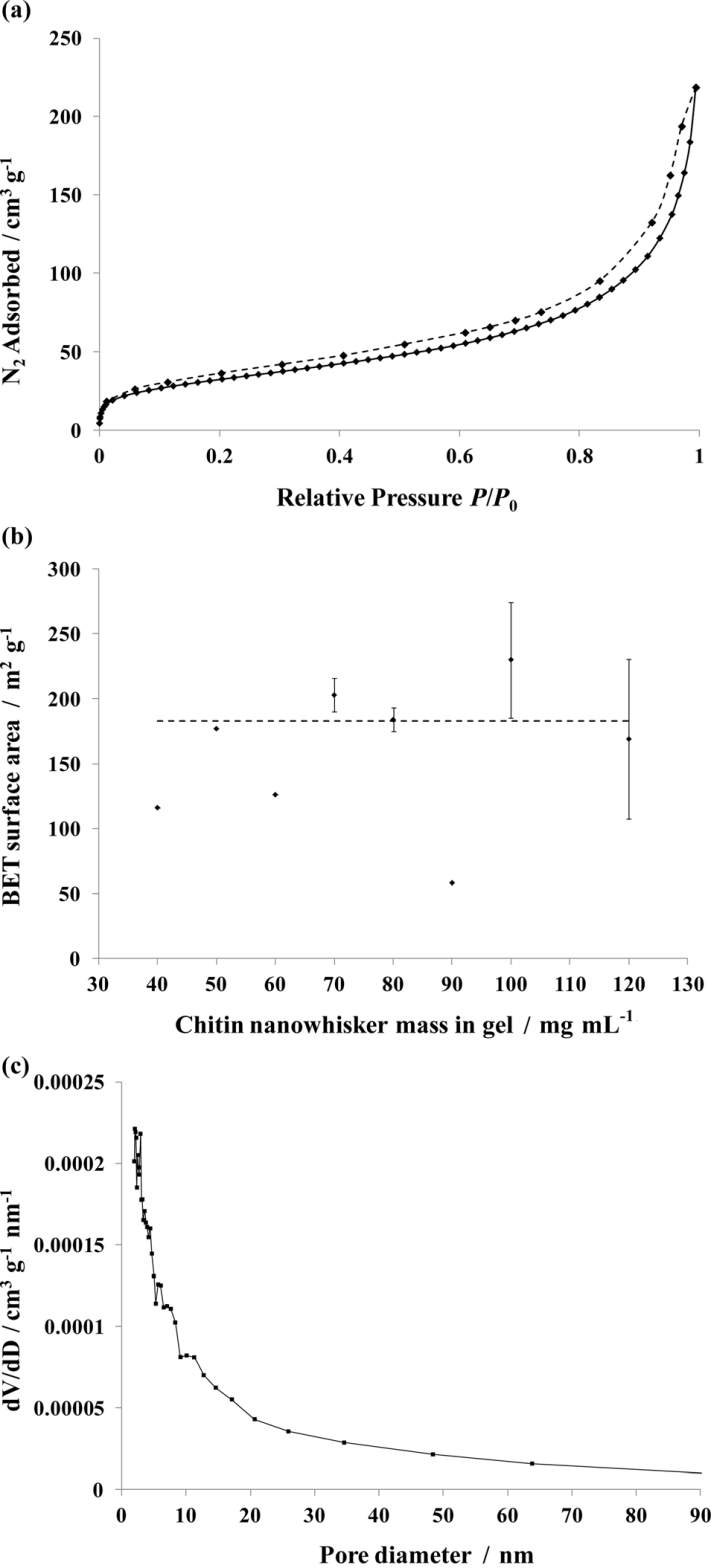
(a) Typical aerogel N_2_ adsorption (—) and desorption isotherm (- - - -), (b) BET surface area shown as a function of the initial chitin nanowhisker mass in the hydrogel. The dotted indicates the calculated specific surface area for the chitin nanowhiskers using average dimensions and assuming no polydispersity, and (c) typical mesopore size distribution for the chitin aerogels.

A representative nitrogen sorption isotherm for our chitin nanowhisker aerogels is shown in Figure 3 a. The isotherms can be classified as Type IV (IUPAC classification), indicating that there is multilayer adsorption on a mesoporous solid. The isotherms display type H3 hysteresis loops that imply the aerogels retain their open mesoporous structure. BET^23^ analysis of the amount of N_2_ adsorbed at *P*/*P*_0_ between 0.06 and 0.35 showed that the BET surface area varied between 58 and 261 m^2^ g^−1^ as shown in Figure 3 b and Table 2, without clear correlation between the initial chitin nanowhisker mass and surface area obtained. The variation between samples is believed to be due to differences in the extent of dispersion during aqueous sonication-gelation step and this is currently under investigation by using small angle neutron scattering experiments.

**Table 2:** The BET surface area is calculated using the amount of N_2_ adsorbed at a relative vapour pressure of 0.06–0.35 at 77 K. *V*_total_ is the total pore volume determined at *P*/*P*_0_ of 0.99; *V*_meso_ is the mesopore volume determined at *P*/*P*_0_ of 0.95; % mesopore volume is the percentage of the total pore volume that is mesoporous; 2*r* is the average pore diameter of the aerogels determined using the adsorption branch of the isotherm, using the BJH method

Initial chitin nanowhisker mass in gel [mg]	BET surface area [m^2^ g^−1^]	*V*_total_ [cm^3^ g^−1^]	*V*_meso_ [cm^3^ g^−1^]	% mesopore volume	2*r* [nm]
40	116	0.34	0.21	62 %	13
50	177	0.49	0.31	63 %	13
60	126	0.33	0.23	70 %	12
70	212	0.64	0.40	63 %	14
70	194	0.66	0.37	56 %	15
80	177	0.69	0.37	54 %	17
80	190	0.64	0.37	58 %	14
90	58	0.19	0.11	58 %	15
100	198	0.60	0.37	62 %	14
100	261	0.75	0.48	64 %	12
120	125	0.47	0.25	53 %	17
120	212	0.68	0.43	63 %	13

The specific surface area was calculated to be 183 m^2^ g^−1^, when average chitin nanowhisker dimensions (240 nm×15 nm×15 nm) determined by Nair et al.,^9^ were used. This value approaches the upper limit of the BET surface areas achieved for these samples. The calculated specific surface area would be correct for an aerogel made up of fully individualized monodisperse chitin nanowhiskers with smooth surfaces. Higher specific surface area values were observed because the chitin nanowhisker were not monodisperse^9^ and their surfaces were not smooth. The lower values can be attributed to chitin nanowhisker aggregation, where alignment of nanowhiskers reduced the surface area accessible for gas adsorption. Therefore, the degree of nanowhisker aggregation has a significant impact on the surface area values obtained. The differences shown between the total pore volume (calculated from aerogel porosity) and mesopore volume of the aerogels indicate that there were a significant number of macropores within the samples (Table 2).

Figure 3 c further shows the pore size distribution of the aerogels calculated from the adsorption branch of the isotherm using Barrett–Joyner–Halenda (BJH)^24^ analysis. The chitin aerogels show a broad size distribution with an average pore size around 14 nm, but with pores spanning the whole mesopore range. In comparison to the cellulose nanowhisker aerogels, the chitin aerogels displayed a wide distribution of mesopores between 2 and 50 nm, with average pore diameters between 12–17 nm, whereas the cellulose nanowhisker aerogels had a bimodal pore size distribution, the reason for which is currently under investigation by neutron scattering experiments. The chitin aerogels displayed smaller surface areas (58–261 m^2^ g^−1^) than the cellulose nanowhisker aerogels (216–605 m^2^ g^−1^)^1^ because the chitin nanowhiskers have a much smaller aspect ratio (16 compared to 30 for cotton-derived cellulose nanowhiskers), and therefore a smaller surface area. The predicted surface area for chitin nanowhisker aerogels is just 183 m^2^ g^−1^ compared to 419 m^2^ g^−1^ for cellulose nanowhisker aerogels (with the cellulose nanowhiskers derived from cotton).

FTIR spectroscopy was utilized to identify the crystal form of the chitin. The presence of three strong absorption peaks in the carbonyl region at 1661, 1644 and 1560 cm^−1^ are characteristic of α-chitin.^25^ The chitin nanowhiskers also comprised pure chitin as indicated by the absence of a peak at 1540 cm^−1^, corresponding to proteins.^26^ The FTIR spectra for the chitin nanowhiskers and the aerogel indicate that the chitin aerogel retained the crystallinity of the chitin nanowhiskers due to the absence of broadening in the O–H stretching region at 3460 cm^−1^ (see Supporting Information).17c

**Figure 4:**
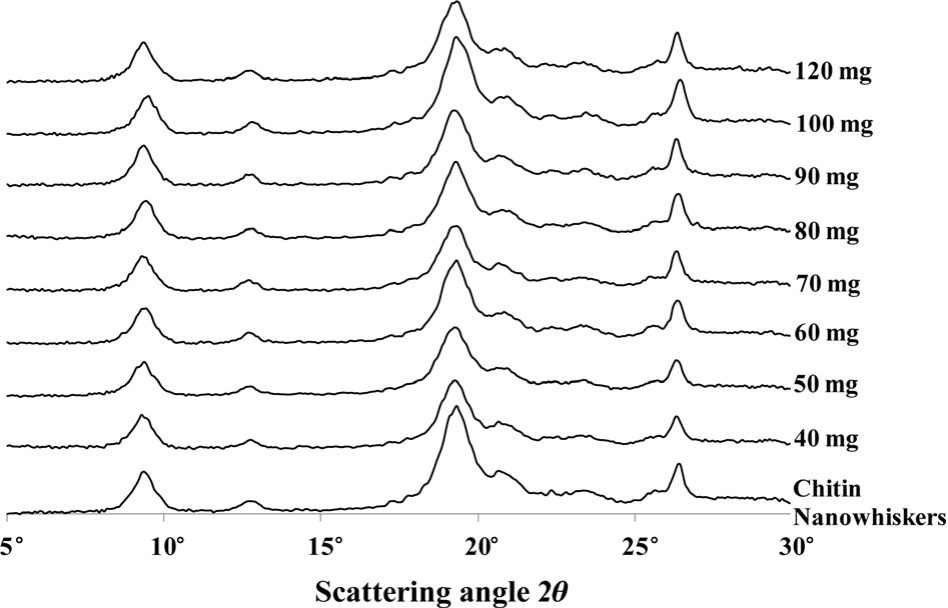
XRD traces versus scattering angle of the chitin nanowhiskers and chitin nanowhisker aerogels.

Powder X-ray diffraction (XRD) was also used to determine the effect of the aerogel preparation on the crystalline structure of the chitin nanowhiskers. Figure 4 compares the diffraction patterns of the chitin aerogels with the diffraction pattern of the chitin nanowhiskers. Diffraction peaks at 9.4°, 12.8°, 19.3°, 20.8°, 23.4°, and 26.4°, corresponding to (020), (021), (110), (120), (130), and (013), respectively, are observed for all samples. These diffraction peaks are consistent with those expected for the α-chitin polymorph.17c, 27 The chitin aerogels clearly retained the α-chitin structure, indicating that neither sonication nor scCO_2_ drying affected the crystalline structure of the chitin nanowhiskers. To quantify the crystallinity shown by the chitin nanowhiskers, the relative crystallinity index (RCI) was calculated by using Equation (1):


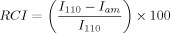
(1)

where *I*_110_ is the intensity, above the baseline, of the 110 peak maximum around 2*θ*=19° and *I*_am_ is the amorphous diffraction at 2*θ*=12.6°.

The RCI of the chitin nanowhiskers is about 86 %, which is in strong agreement with literature values.^28^ The RCI was consistent for all aerogel samples. This confirms that the sonication step and the scCO_2_ drying step did not affect the crystalline structure of the chitin nanowhiskers. The aerogels were therefore a true 3D assembly of chitin nanowhiskers. The retention of the chitin crystalline nature is believed to be important to achieve the low shrinkage rates seen for these aerogels (6.5 % for cellulose nanowhiskers and 4 % for chitin nanowhiskers), since the rigidity of the nanocrystals prevents buckling, making slippage between hydrogen-bonded nanowhiskers the only shrinkage mechanism during drying.

Thermogravimetric analysis (TGA) of the aerogel samples showed that the decomposition profiles of the chitin nanowhiskers and aerogels are consistent, with small weight losses below 150 °C (as shown in Table 3) attributed to the loss of adsorbed water (see Supporting Information). The differential TGA curves indicate that the aerogel decomposed at a rate consistent with the chitin nanowhiskers, resulting in the aerogels retaining the thermal stability of the chitin nanowhiskers.

**Table 3:** Percentage mass loss at 150 °C for the chitin nanowhiskers and chitin aerogels

Sample	Mass loss at 150 °C
Chitin nanowhiskers	7.2 %
40 mg aerogel	5.1 %
50 mg aerogel	5.3 %
60 mg aerogel	5.3 %
70 mg aerogel	5.7 %
80 mg aerogel	5.4 %
90 mg aerogel	5.5 %
100 mg aerogel	5.7 %
120 mg aerogel	4.5 %

**Figure 5:**
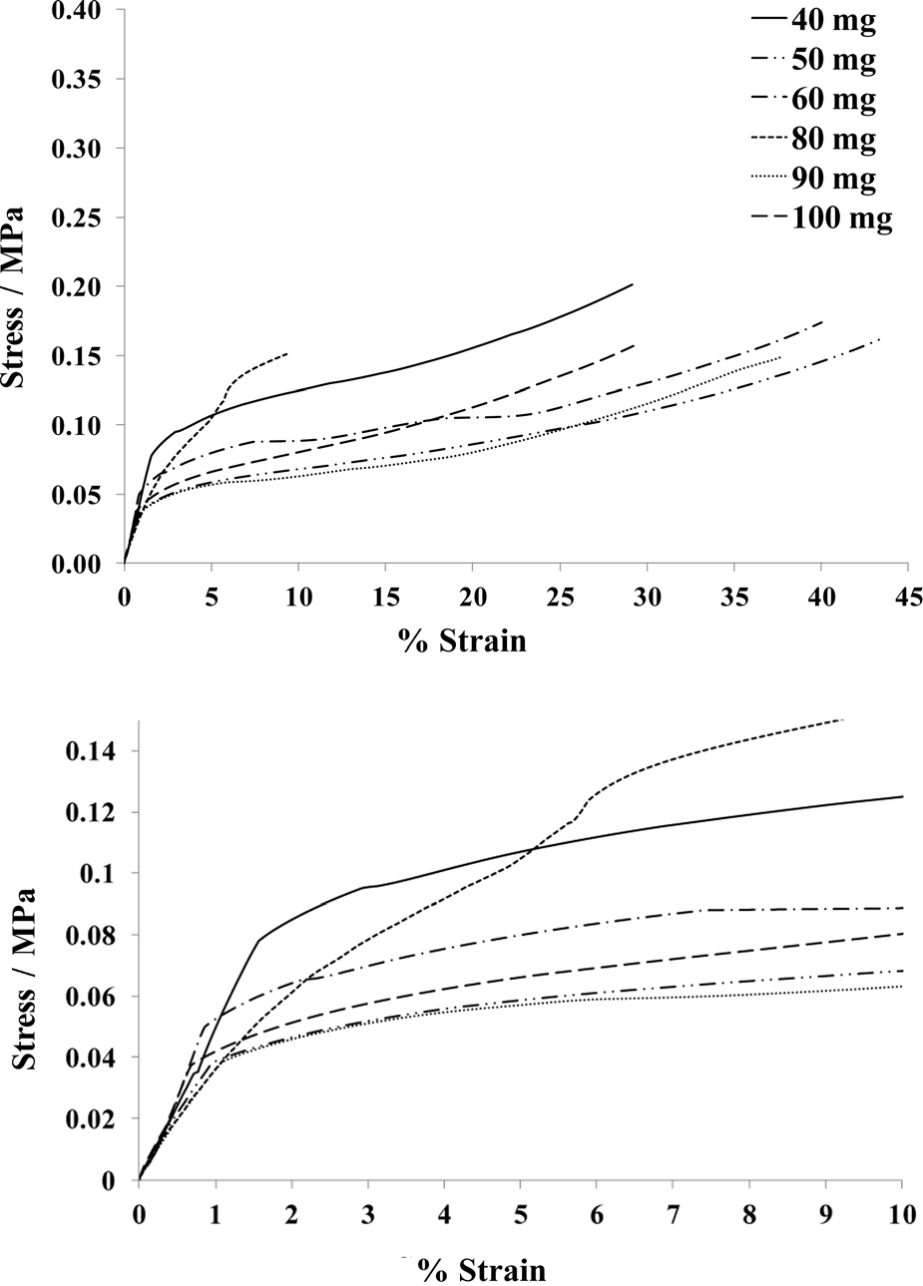
(a) Compression stress–strain curves of chitin nanowhisker aerogels, (b) Compression stress–strain curves at low strain.

Compression measurements were performed to quantify the aerogels’ mechanical properties, shown in Table 4. The reported specific compressive modulus is the Young’s modulus divided by the density of the aerogel, enabling cross-comparison between aerogels of different densities. The stress–strain curves (Figure 5) show an initial linear slope at small strain values, indicating the elastic behavior of the samples. The Young’s modulus was calculated from the initial linear region. The Young’s modulus remained fairly consistent around 7.2 MPa for all of the samples with variable density between 0.052 and 0.098 g cm^−3^. Only the least dense aerogel (0.043 g cm^−3^) showed a higher modulus of 9.3 MPa. The specific compressive modulus (adjusted for density differences) showed a consistent decrease with increasing density, signifying a decreasing strength contribution with increasing nanowhisker addition. This implies increased aggregation of the nanowhiskers, as longitudinal alignment of the nanowhiskers reduces their individual contribution to the percolated network, leading to a lower reinforcement per nanowhisker.

**Table 4:** Mechanical properties of various chitin nanowhisker aerogels with different densities

Sample	Density [g cm^−3^]	Young’s modulus [MPa]	Specific compressive modulus [×10^6^ m^2^ s^−2^]
40 mg aerogel	0.043	9.32	0.217
50 mg aerogel	0.052	7.60	0.146
60 mg aerogel	0.063	7.57	0.120
80 mg aerogel	0.080	7.30	0.091
90 mg aerogel	0.092	7.10	0.077
100 mg aerogel	0.098	7.07	0.072

After the linear region, permanent deformation of the aerogels occurred before the porous structure completely collapsed and significant densification of the material began. The Young’s modulus achieved for the chitin aerogels (9.32–7.07 MPa) significantly improves upon those reported for polymer/clay aerogels^29^ (with a density of 0.07 g cm^−3^ and a modulus of 0.23 MPa) and polymer/clay/nanotube aerogels^30^ (with a density of 0.05 g cm^−3^ and a modulus of 0.63 MPa). Our chitin aerogels also displayed a higher modulus than microfibrillated cellulose aerogels19c of a similar density range (0.007–0.103 g cm^−3^) with a reported Young’s modulus range of 0.056–5.31 MPa. Aerogels with higher densities, such as clay/cellulose nanowhisker aerogels^31^ (0.101 g cm^−3^), polymer/silica aerogel^32^ (0.11 g cm^−3^), and clay/polymer aerogels^33^ (0.2 g cm^−3^) also displayed lower Young’s modulus values than our chitin aerogels. More recently, Ding and co-workers reported higher Young’s modulus values (72.5–76.2 MPa) than those found for our chitin aerogels, however, their chitin aerogels had significantly larger densities (0.23–0.27 g cm^−3^) giving similar specific compressive moduli as reported here in the range of 0.28–0.32×10^6^ m^2^ s^−2^.

**Figure 6:**
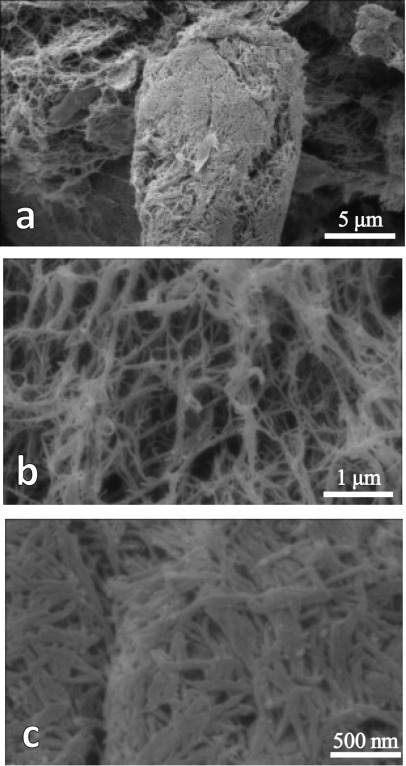
SEM images of a representative 120 mg chitin aerogel where (a) shows the porous network and aggregation of chitin nanowhiskers, (b) shows the highly porous network at higher magnification, and (c) shows how the aggregated chitin nanowhiskers are arranged in bundles.

Scanning electron microscopy (SEM) was used to image the internal porous network structure of the aerogels. Figure 6 shows representative images taken of a 120 mg aerogel at three different magnifications. The highly porous network is clearly visible in Figure 6 a and b with aggregation of the nanowhiskers into bundles shown in Figure 6 a and c. The pores within the bundles were in the sub-500 nm size range (Figure 6 c), consistent with the BET results whilst Figure 6a shows that a significant amount of macropores also exist in the structure. The aggregated bundles of nanowhiskers decrease the specific surface area of the aerogel. This is reflected in the BET surface area value of 125 m^2^ g^−1^ for this specific sample, which is significantly lower than the predicted 183 m^2^ g^−1^ if all of the nanowhiskers are well dispersed.

## Conclusions

Mesoporous chitin nanowhisker aerogels were prepared by sonication-assisted assembly of chitin nanowhiskers in water followed by solvent exchange with ethanol and drying with scCO_2_. These aerogels were highly porous, with low densities and moderate surface areas. As indicated by FTIR and XRD, the chitin nanowhiskers retained their crystalline structure throughout the production process confirming that the aerogels were 3D assemblies of chitin nanowhiskers. Shrinkage during drying was found to be extremely limited at only 4 %. The aerogels also retained the thermal stability of the chitin nanowhiskers which could prove invaluable in many applications, and also displayed mechanical properties in the upper range of other reported aerogels. These aerogels were made by using a simple and benign process, resulting in their ability to find potential application in a wide variety of areas, such as thermal insulators, catalyst supports, and biomedical materials.

## Experimental Section

**Safety Note**: Experiments with scCO_2_ involve high pressures and should only be carried out in equipment with the appropriate pressure rating and safety operating procedures.

All chemicals were used as purchased from Fisher Scientific, Sigma–Aldrich, and Alfa Aesar and used as received unless otherwise stated.

Densities of the samples were determined by weighing the monoliths on an analytical balance (0.01 mg accuracy) and measuring their dimensions by using a digital caliper (±0.02 mm). Dimensions for each aerogel sample were taken at five different positions. Percentage porosity of the sample (*P*) was calculated using the density of the samples (*d*_p_) and the density of the bulk chitin nanowhiskers (*d_b_*=1.5 g cm^−3^), obtained from the simple mixing rule where the gas density is negligible as it is more than 1000 times lower.


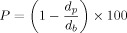
(2)

Powder XRD data was recorded on a PANalytical X′Pert Pro MPD in Bragg–Brentano geometry, with monochromated Cu K_α1_ (*λ*=1.5406 Å, 40 kV, 40 mA) radiation, automated divergence and receiving slits (10 mm illuminated length), 10 mm beam mask, 0.04 rad soller slits, and a step size of 0.08°. Nitrogen physisorption measurements were carried out by using a Micrometrics ASAP 2020 at 77 K. BET and BJH analyses were completed using Datamaster software. The samples were degassed at 80 °C in vacuum in order to remove all of the adsorbed species. The BET analysis was performed for the relative vapor pressures corresponding to a meso-porous adsorbent of 0.05–0.35, where the straight line fit was verified for each sample. The BJH analysis was performed using the adsorption branch of the isotherms.

SEM analysis was carried out using a Philips XL30 FEG Environmental scanning electron microscope operating at 20 kV. TEM images of chitin nanowhiskers were recorded on an JEOL JEM 2000 FXII electron microscope operating at an acceleration voltage of 80 kV. A carbon-coated Cu grid was treated under a 25 % oxygen in argon plasma for 5 s. A suspension of nanowhiskers was deposited on the grid and left for 3 min after which excess liquid was removed. The grid was then stained using a 2 % uranyl acetate in water solution for 5 min and dried before analysis.

FTIR spectra were recorded on a Thermo Nicolet 380 spectrometer using KBr pellets made with a weight ratio of sample to KBr of 1:99, in the region 400–4000 cm^−1^. TGA was performed on a TA Instruments TGA Q500, using approximately 5–15 mg of sample in an open aluminum pan. Compressed air was used as the purge gas and the flow rate was 40 cm^3^ min^−1^ through the furnace and 60 cm^3^ min^−1^ through the TGA head. The samples were heated from 25 °C to 600 °C with an applied scanning rate of 10 °C min^−1^. Universal Analysis software (TA Instruments) was used for the analysis of all TGA results. Compression strengths were measured using a TA Instruments Q800 DMA analyzer using parallel plate compression clamps. The stress/strain controlled force mode was used. The samples used in the compression tests were between 5 and 9 mm thick, the force ramp rate was 2 N min^−1^, and the maximum loading force was 18 N. All samples were kept under isothermal conditions at 25 °C for 5 min before the measurement and the temperature remained constant throughout the measurement. The Young’s modulus was calculated from the initial linear region of the stress-strain curves.

### Preparation of chitin nanowhiskers

Chitin nanowhiskers were obtained from crab shells in a procedure described previously by Nair and Dufresne.^9^ Briefly, chitin was boiled and stirred in a 5 % KOH solution for 6 h to remove most of the proteins. The suspension was then kept at room temperature overnight under stirring and subsequently filtered and washed with deionized water. The chitin was then bleached with NaClO_2_ (17 g) in deionized water (1 L) containing 0.3 M sodium acetate buffer for 6 h at 80 °C. The bleaching solution was changed every two hours followed by abundant rinsing of the sample with de-ionized water. After bleaching, the solutions were kept in a 5 % KOH solution for 48 h. The resulting suspension was then centrifuged at 10 000 rpm for 15 min.

The purified chitin was subjected to acid hydrolysis to yield the chitin nanowhiskers by boiling in 3 N HCl for 90 min under stirring. The ratio of 3 N HCl to chitin was 30 cm^3^ g^−1^. After hydrolysis, the chitin nanowhiskers were washed with deionized water and centrifuged for 15 min at 10 000 rpm. The supernatant was discarded and the nanowhiskers were redispersed in deionized water and centrifuged again. This process was repeated three times. The nanowhiskers were dialyzed against tap water for 2 h and then overnight in deionized water to remove any free acid. Nanowhiskers were redispersed by sonication using a Branson sonifier for 5 min, in 3 s pulses with 2 s intervals at an amplitude of 15 % of the maximum power, with a maximum temperature of 30 °C. Aggregated nanowhiskers were removed by filtration over a No. 2 fritted filter. Amberlite MB 6113 was added under agitation for 1 h in a ratio of roughly 10 g L^-1^ of nanowhisker suspension to replace all cations on the surface of the chitin nanowhiskers with protons. This ion exchange resin also displays a color change upon saturation so it was verified that the ion exchange capability was not saturated. The Amberlite MB was removed by filtration and the dispersion was redispersed by sonication for five minutes. The dispersion was plunged into liquid nitrogen to freeze before being attached to a Heto PowerDry LL3000 freeze dryer until the nanowhiskers were completely dry.

### Aerogel preparation

Freeze dried chitin nanowhiskers were used to produce aerogels in a three step method as described in our previous publication for cellulose nanowhiskers.^1^ Hydrogels with varying initial chitin nanowhisker mass were produced by dispersing various amounts of nanowhiskers in 1 mL deionised water at 25 °C using a low power sonication bath (Sonomatic 375 Ultrasonic Cleaner, Agar Scientific). Sonication was performed in 15 min intervals until the samples gelled. The point of gelation was determined as the point at which the vial could be inverted without net movement of the gel. Once formed, the hydrogels underwent a solvent exchange procedure in excess anhydrous ethanol, changing the ethanol every day for four days at room temperature. The alcogels were subsequently dried under scCO_2_ by using an in-house built flow-through autoclave at 40 °C and 100 bar. The CO_2_ flow rate was kept constant at 2 mL min^−1^ for 6 h to ensure that all of the ethanol was completely removed.

## Supplementary material

As a service to our authors and readers, this journal provides supporting information supplied by the authors. Such materials are peer reviewed and may be re-organized for online delivery, but are not copy-edited or typeset. Technical support issues arising from supporting information (other than missing files) should be addressed to the authors.
