# Supplementary material for: Chitin Nanowhisker Aerogels
Source: ChemSusChem. 2013 Jan 18;6(3):537–44. doi: 10.1002/cssc.201200717 (PMC3615177; doi:10.1002/cssc.201200717)
Supplement: Supplementary file 1 [file cssc0006-0537-SD1.pdf]

CHEMISTRY & SUSTAINABILITY
CHEMSUSCHEM
ENERGY & MATERIALS

Supporting Information

© Copyright Wiley-VCH Verlag GmbH & Co. KGaA, 69451 Weinheim, 2013

Chitin Nanowhisker Aerogels

Lindy Heath,[a] Lifan Zhu,[b] and Wim Thielemans*[a, b]

cssc_201200717_sm_miscellaneous_information.pdf

1. Infrared Spectra

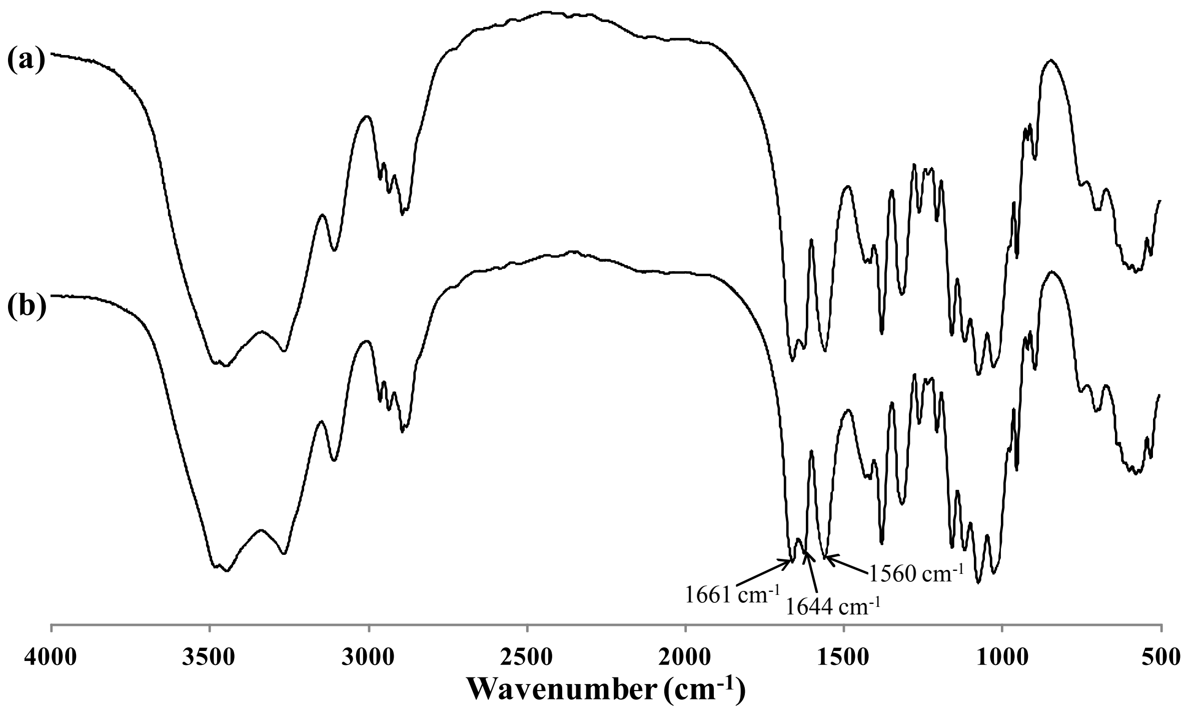

Figure ESI-1 FTIR spectrum of (a) chitin nanowhiskers and (b) chitin nanowhisker aerogel.

2. Thermogravimetric Analysis

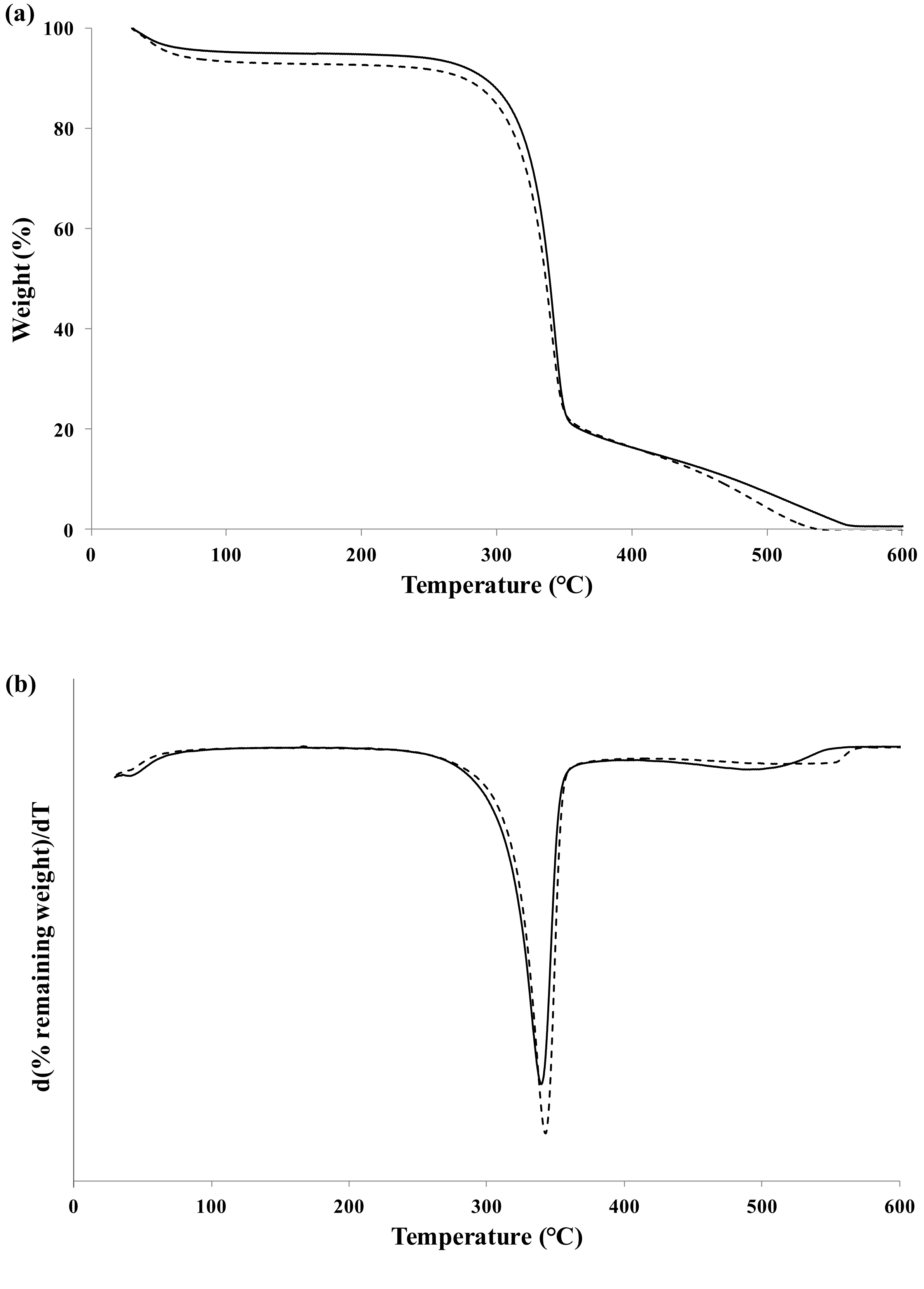

Figure ESI-2 (a) TGA curves of chitin nanowhiskers (----) and chitin aerogel (80 mg/ mL) (—) and (b) differential TGA curves of chitin nanowhiskers (----) and chitin aerogel (80 mg/ mL) (—).
